# Atomoxetine for Intradialytic Hypotension in a Patient on Hemodialysis: A Case Report

**DOI:** 10.1016/j.xkme.2024.100840

**Published:** 2024-05-17

**Authors:** Yi-Hsin Chen, Chih-Tsung Chen

**Affiliations:** 1 Department of Nephrology, Taichung Tzu Chi Hospital, Taichung, Taiwan; 2 School of Medicine, Tzu Chi University, Hualien, Taiwan; 3 Department of Artificial Intelligence and Data Science, National Chung Hsing University, Taichung, Taiwan

**Keywords:** Atomoxetine, autonomic dysfunction, hemodialysis, intradialytic hypotension (IDH)

## Abstract

Intradialytic hypotension significantly affects patient safety and clinical outcomes during hemodialysis. Despite various pharmacological and nonpharmacological interventions, effective management remains elusive. In this report, we detail a case of intradialytic hypotension in a male patient in his 40s, undergoing hemodialysis with a history of polycystic kidney disease. Eight years ago, the patient underwent bilateral nephrectomy because of a severe cystic infection, after which his systolic blood pressure (BP) persistently remained at 50-70 mm Hg during dialysis sessions. The initial treatment strategy for hypotension included fludrocortisone, midodrine, and prednisolone, leading to a slight temporary increase in BP, which subsequently declined. As the patient’s condition deteriorated, the administration of norepinephrine or dopamine became necessary to sustain BP during dialysis. Given the patient’s resistance to these treatments, a daily dose of 25 mg of atomoxetine was introduced. Following this treatment, there was a gradual improvement in the patient’s vertigo, weakness, and BP. This case illustrates that low-dose atomoxetine can alleviate symptoms and elevate BP in patients experiencing severe intradialytic hypotension during hemodialysis.

## Introduction

Intradialytic hypotension (IDH) presents a significant challenge for hemodialysis (HD) patients. The National Kidney Foundation’s Kidney Disease Outcomes Quality Initiative defines IDH as a decline in systolic blood pressure (SBP) exceeding 20 mm Hg, often accompanied by symptoms such as cramping, vomiting, or chest pain.^1^ A study by Flythe et al^2^ further associates a nadir SBP below 90 mm Hg with an increased risk of mortality. The prevalence of IDH based on various definitions ranges from 8%-14%. It can disrupt dialysis therapy and lead to fluid accumulation, potentially resulting in cardiac arrest.

IDH’s complex pathophysiology makes prevention and treatment difficult. A decrease in blood pressure (BP) during dialysis is typically related to blood volume reduction postultrafiltration; other identified risk factors include gender, diabetes, and age at dialysis.^3^^,^^4^ The study by Tislér et al^5^ highlights that pre-existing coronary heart disease can also induce IDH, whereas an analysis by Shafi et al^6^ indicates autonomic dysfunction contributes to IDH during ultrafiltration failure. Modifiable factors affecting IDH include dialysate temperature, sodium profiling, and the ultrafiltration rate.^7^

Various pharmacologic and nonpharmacologic interventions have been explored for IDH. Pharmacologic treatments like midodrine and L-carnitine have shown inconclusive effectiveness.^8^^,^^9^ Nonpharmacologic approaches, such as cool dialysate and low ultrafiltration rate, have been either ineffective or problematic.^10^, ^11^, ^12^

Atomoxetine, a Food and Drug Administration-approved norepinephrine transporter blocker for attention deficit hyperactivity disorder, has also demonstrated efficacy in treating orthostatic hypotension in several studies.^13^^,^^14^ However, its application in treating IDH among HD patients remains unexplored, marking this case study as a novel investigation.

## Case Report

The patient, a male in his 40s with adult polycystic kidney disease, had been on maintenance HD for 10 years. His brother also had a history of HD for adult polycystic kidney disease. The patient underwent bilateral nephrectomy because of cyst infection and subsequent septic shock 9 years prior. During both interdialytic and intradialytic periods, he consistently experienced low BP, with systolic BP readings between 70 and 80 mm Hg, occasionally dropping to 50 mm Hg postdialysis.

Initially, the patient was prescribed 5 mg midodrine thrice daily and 0.1 mg fludrocortisone twice daily for low BP, which yielded partial effectiveness; symptoms of dizziness and fatigue were temporarily alleviated. However, a year before the current hospital admission, his symptoms exacerbated. His BP during interdialytic periods were decreased to 70-80 mm Hg, and predialysis systolic BP often dropped to around 60 mm Hg, creating a critical situation. Subsequently, he was admitted to the intensive care unit because of this severe hypotension. To manage the dizziness and low BP, vasopressors such as dopamine and norepinephrine were administered.

He experienced hypotension postdialysis in late December 2021, where neither rest nor rehydration sufficiently improved his BP. In the hospital ward, he initially received 6 mL/hour of norepinephrine for a BP of 63/33 mm Hg while supine. This dosage was increased to 12 mL/hour because of persistent low BP, resulting in an improved BP of 102/54 mm Hg over 20 hours. A comprehensive evaluation was conducted to identify the cause of his consistently low BP. Tests including serum thyroid stimulating hormone, adrenocorticotropic hormone, morning cortisol, aldosterone, and renin levels yielded results within normal ranges of 1.03 μIU/mL, 3.55 pg/mL, 25.07 μg/dL, 15.5 ng/mL, and 0.07 ng/mL/h, respectively. Transthoracic echocardiography showed normal left ventricular function, with an ejection fraction of 70.4% and concentric left ventricular wall hypertrophy.

Additionally, a consulting neurologist identified dysautonomia and conducted sympathetic skin response and R-R interval variation studies. Given the ineffectiveness of previous treatments for his severe hypotension, cortisone (50 mg twice a day) was introduced. However, managing his BP continued to be challenging. As a result, atomoxetine 25 mg daily was prescribed, leveraging its proven efficacy in treating orthostatic hypotension in previous studies.^13^^,^^15^^,^^16^ This treatment plan was responsive to the patient’s evolving clinical needs. In selecting this patient for atomoxetine, we prioritized his recurrent IDH episodes and the ineffectiveness of conventional treatments. Norepinephrine was gradually tapered off once his BP stabilized at 100/60 mm Hg. On discharge, his medication regimen included 50 mg cortisone twice daily, 0.1 mg fludrocortisone twice daily, 5 mg midodrine 3 times daily (as previously taken), and atomoxetine 25 mg daily.

During the December 2021 to January 2022 follow-up, the patient completed 15 dialysis sessions. In these sessions, he remained alert but occasionally experienced dizziness and palpitations following atomoxetine administration, without necessitating any dialysis session discontinuation. The lowest interdialytic BP reading without fatigue or muscle cramps was recorded at 69/48 mm Hg.

**Figure 1:**
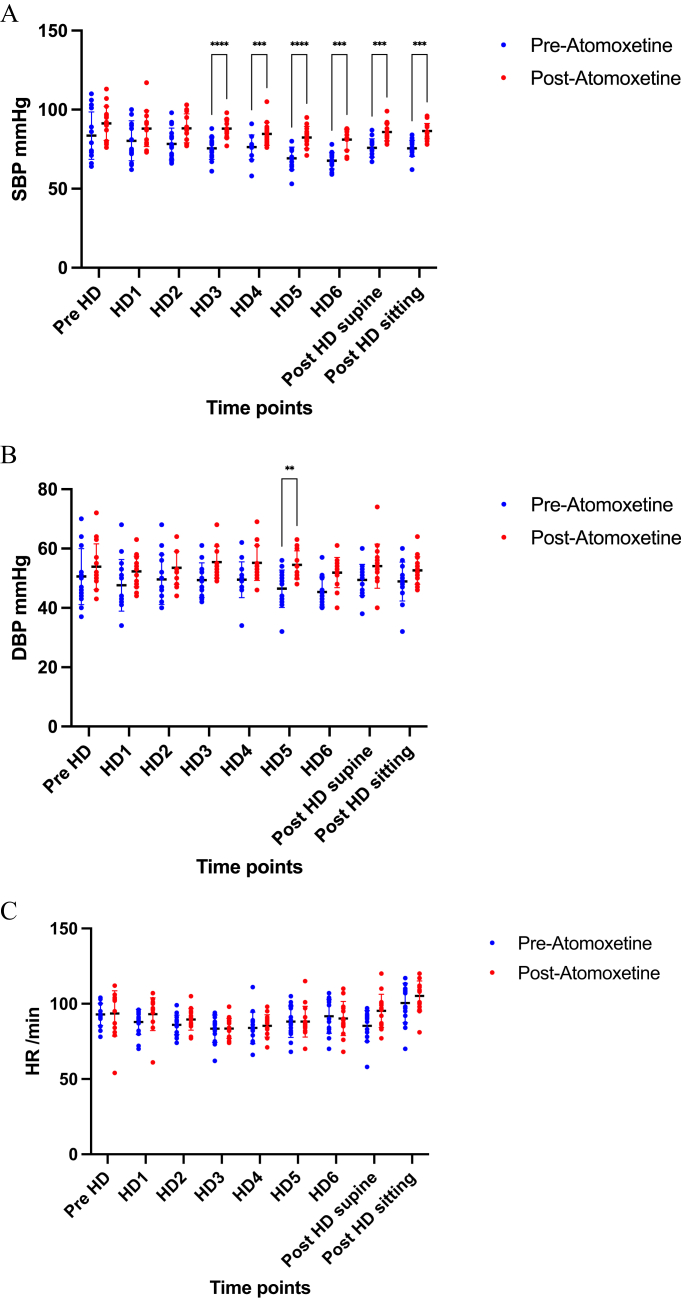
Pre- and postatomoxetine hemodynamic changes at different timepoints. (A) Changes in systolic blood pressure, (B) diastolic blood pressure, and (C) heart rate. HD1-HD6 represents readings at 0, 60, 120, 180, 210, and 240 minutes following the initiation of dialysis therapy, respectively. DBP, diastolic blood pressure; HR, heart rate; SBP, systolic blood pressure.

**Figure 2:**
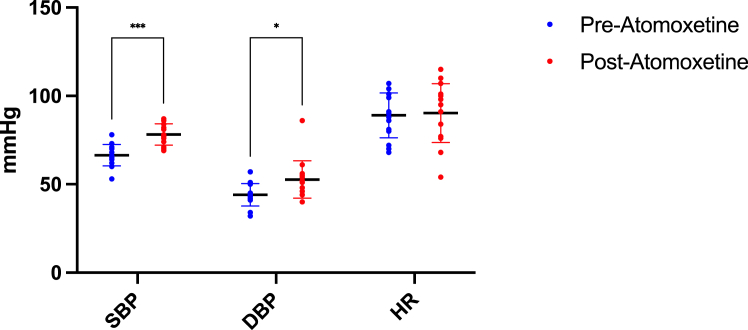
Pre- and postatomoxetine changes in nadir systolic blood pressure, diastolic blood pressure, and heart rate. Significant differences observed in nadir SBP and DBP. DBP, diastolic blood pressure; HR, heart rate; SBP, systolic blood pressure.

The hemodynamic changes before and after atomoxetine treatment are depicted in Figures 1 and 2. Pre-HD denotes predialysis sitting BP, whereas HD1-HD6 represents BP readings at various time intervals (0, 60, 120, 180, 210, and 240 minutes) postdialysis initiation. Post-HD supine and Post-HD sitting refer to BP readings 5 minutes postdialysis in respective positions. Notably, Figure 1A illustrates a significant increase in systolic BP from HD3 to HD5 following atomoxetine administration compared with preatomoxetine levels. This trend contrasts with the gradual decrease observed in intradialytic systolic BP before atomoxetine use, which was consistently maintained posttreatment. At HD5, there was an average increase of 13.33 ± 2.08 mmHg in BP after atomoxetine administration. Figure 1B shows a significant increase in diastolic BP on HD5 after atomoxetine treatment compared with the pretreatment levels. Figure 1C indicates no significant variation in heart rates before and after atomoxetine administration.

During HD sessions, nadir SBP readings after atomoxetine administration exhibited an increase compared to pre-atomoxetine levels (Fig 2). The associated diastolic BP readings with these nadir SBP values averaged at 52.73 ± 10.53 mmHg. Fluid removal volumes during HD sessions remained consistent before and after atomoxetine treatment. Liver function tests posttreatment indicated normal results. At home, the mean sitting BP post-atomoxetine averaged 99.48/64.90 mmHg, and the mean supine BP was 100.14/63.51 mmHg. After 6 months of atomoxetine therapy, the patient independently ceased medication for approximately 1 week because of unintentional nonadherence, while continuing his cortisone, fludrocortisone, and midodrine regimen. This led to a notable decrease in SBP to around 70 mm Hg during dialysis, complicating the procedure. Concurrent home BP readings also declined, with SBP nearing 90 mm Hg. Recognizing the detrimental effect on BP control, the patient promptly resumed atomoxetine at 25 mg daily, effectively restoring BP to previously stabilized levels achieved during therapy.

## Discussion

This case report examines the effectiveness of atomoxetine in managing in IDH among HD patients, providing new insights into potential therapeutic approaches. A study demonstrated that 18 mg of atomoxetine significantly increased BP compared to a placebo in 21 patients with central autonomic failure.^17^ Furthermore, Byun et al^15^ reported that atomoxetine, unlike midodrine, ameliorated symptoms related to orthostatic hypotension in a 1-month follow-up. In this case, the patient also experienced an improvement in IDH-related dizziness. Notably, the most significant change was an increase in BP during HD sessions.

Within the nephrology field, IDH has commonly been treated with midodrine.^18^ However, the Food and Drug Administration considered withdrawing midodrine from the market in 2010, citing insufficient evidence of its efficacy based on postmarket evaluations.^19^ Additionally, another study indicated that midodrine did not significantly improve clinical or hemodynamic outcomes.^8^ Despite this, the role of midodrine in IDH treatment remains a subject of debate. For the patient in this case report, midodrine proved ineffective before the introduction of atomoxetine treatment.

Atomoxetine, recognized as a selective norepinephrine reuptake inhibitor, initially gained approval for treating attention deficit hyperactivity disorder. Its efficacy in managing orthostatic hypotension is linked to its ability to increase norepinephrine at synapses, thereby aiding in countering the hemodynamic instabilities.^13^^,^^15^^,^^16^ In this particular case, the patient experienced a notable improvement in BP stability following the initiation of atomoxetine therapy. Importantly, a significant recurrence of hypotensive symptoms was observed on discontinuing atomoxetine, which subsided once the treatment was resumed. This pattern highlights atomoxetine’s effectiveness in managing IDH in this patient, surpassing the results of cortisone and other therapies.

The specific response to stopping and restarting atomoxetine is pivotal, providing concrete evidence of its role in stabilizing BP. This direct correlation between the use of atomoxetine and BP improvement, especially following limited success with other treatments including cortisone, bolsters its potential as a viable treatment option for IDH. During treatment, the patient experienced some side effects, such as dizziness and palpitations, which were closely monitored and conservatively managed, without needing to discontinue atomoxetine. Reflecting on the broader application of these findings to the HD patient population with IDH, it is important to consider the limitations in extrapolating results from a single case study. Although our patient responded positively to atomoxetine, this outcome may not be universally applicable to all IDH patients because of individual health differences. Thus, extensive studies, particularly randomized controlled trials, are necessary to conclusively determine the efficacy and safety of atomoxetine for a broader spectrum of IDH treatment. The study by Byun et al^15^ supports the superior performance of atomoxetine over midodrine in treating orthostatic hypotension. Our case report endorses the use of atomoxetine for IDH patients on HD. Though our findings are promising in the short term, the long-term implications for HD patients warrant further investigation. Comprehensive prospective randomized trials are essential to validate the long-term efficacy and safety of atomoxetine in the HD setting.

In summary, atomoxetine emerges as a potential alternative treatment for IDH in HD patients, particularly when standard therapies are ineffective. Health care professionals should consider low-dose atomoxetine as a viable and safe option in these cases. This study’s findings contribute valuable insights to the management of IDH, offering guidance for clinicians in optimizing patient care.
